# Supplementary material for: Mutations in PCYT2 disrupt etherlipid biosynthesis and cause a complex hereditary spastic paraplegia
Source: Brain. 2019 Oct 22;142(11):3382–97. doi: 10.1093/brain/awz291 (PMC6821184; doi:10.1093/brain/awz291)
Supplement: awz291_Supplementary_Data [file awz291_supplementary_data.zip › awz291-Suppl_data/Supplementary_Data4.zip!/Supplementary information 4 - plasma lipidomics.zip!/Plasma lipidomics/Control vs Patient3b/Statistical plots and data/PCA.pdf]

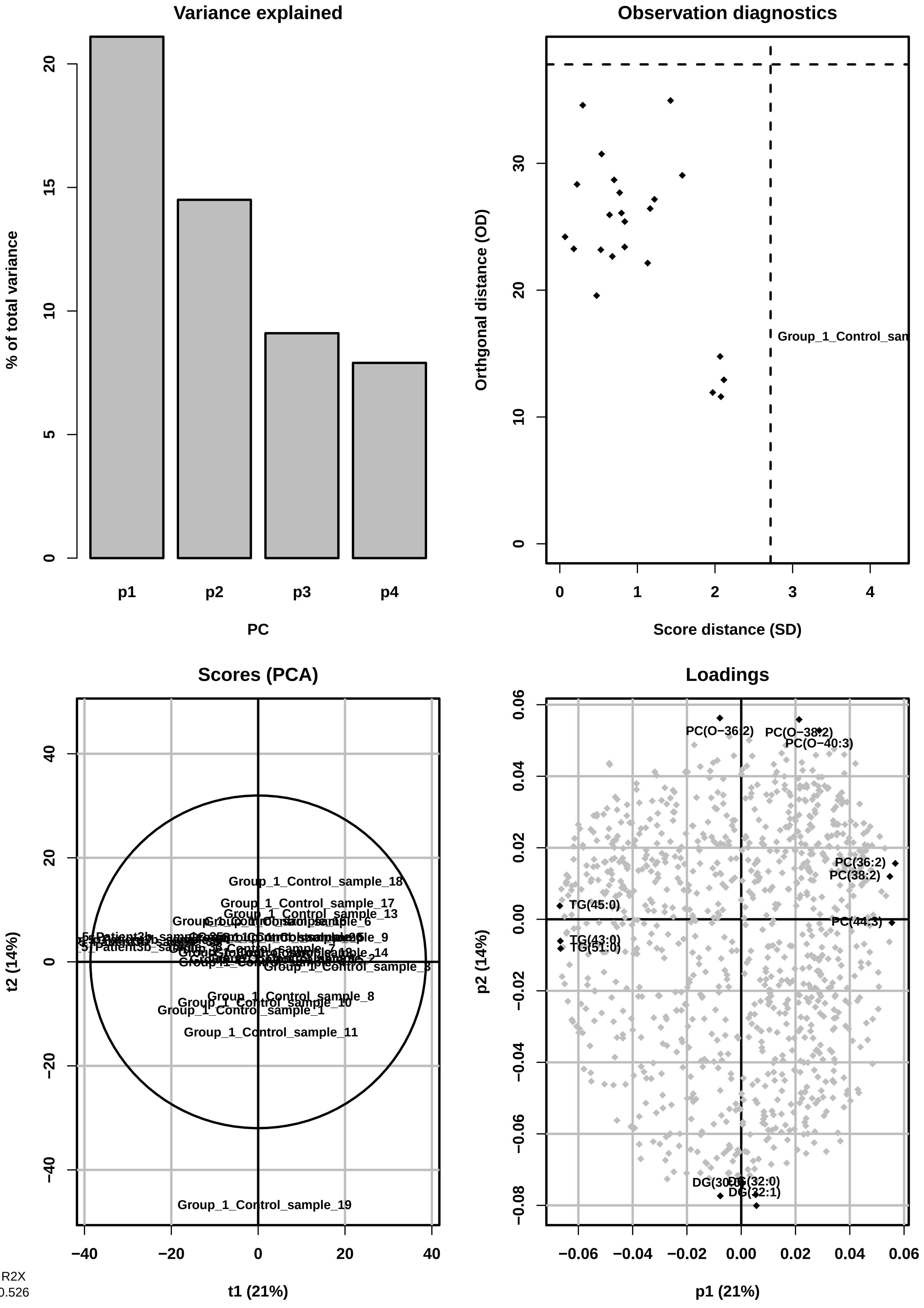
Variance explained
% of total variance
0
5
10
15
20
p1
p2
p3
p4
PC
Observation diagnostics
Orthgonal distance (OD)
0
10
20
30
Group_1_Control_san
0
1
2
3
4
Score distance (SD)
Scores (PCA)
Group_1_Control_sample_18
Group_1_Control_sample_17
Group_1_Control_sample_13
Group_1_Control_sample_3
Group_1_Control_sample_8
Group_1_Control_sample_10
Group_1_Control_sample_1
Group_1_Control_sample_11
Group_1_Control_sample_19
t2 (14%)
t1 (21%)
-40
-20
0
20
40
Loadings
PC(O−36:2)
PC(O−38:2)
PC(O−40:3)
PC(36:2)
PC(38:2)
PC(44:3)
TG(45:0)
TG(43:0)
TG(51:0)
DG(32:0)
DG(32:1)
p2 (14%)
p1 (21%)
−0.08
−0.06
−0.04
−0.02
0.00
0.02
0.04
0.06
R2X
0.526
